# Attitudes of Victim-Blame Toward Image-Based Sexual Abuse Victim-Survivors in Fiji: Understanding Gendered and Cultural Norms

**DOI:** 10.1007/s10508-026-03467-5

**Published:** 2026-07-02

**Authors:** Asher Flynn, Emma Quilty, Natasha Khan, Soropepeli Ramacake

**Affiliations:** 1 Australian Research Council Centre of Excellence for the Elimination of Violence Against Women, Monash University, School of Social Sciences, 10 Ancora Imparo Way, Clayton, VIC 3800 Australia; 2 Australian Research Council Centre of Excellence for the Elimination of Violence Against Women, University of South Pacific, Suva, Fiji Islands

**Keywords:** Image-based sexual abuse, Fiji, Technology-facilitated sexual violence, Patriarchal norms, Sexual taboos, Bulubulu

## Abstract

Victim-blaming and entrenched gender norms are a persistent barrier to justice and support pathways for victim-survivors of sexual and domestic violence in Fiji. While these issues have been considered in relation to physical forms of abuse, there remains limited understanding of how these attitudes may impact victim-survivors of technology-facilitated sexual violence, particularly image-based sexual abuse—the non-consensual creation, capture, distribution, or threatened distribution of nude or sexual imagery. This article examined these dynamics drawing on a reflexive thematic analysis of focus groups run with 30 young people aged 18–24 years across the Fijian cities of Suva, Labasa, and Lautoka. The focus groups explored young people’s attitudes toward image-based sexual abuse victimization, including using the illustrative case study of former Fijian Minister for Women, Children and Social Protection, Lynda Tabuya, who had a private sexual video of her disseminated online without her consent and was subsequently dismissed from her Ministerial position. Our paper explores how cultural, religious, and moral frameworks shape perceptions of victimhood, purity, and blame among young Fijians. Our findings reveal that patriarchal and moralistic narratives contributed to a hostile environment for image-based sexual abuse victim-survivors, where they are often silenced and held responsible for their abuse, while perpetrators evade accountability. This research contributes to the growing literature on gender-based violence in Fiji and underscores the need for culturally grounded education and awareness initiatives that challenge victim-blaming and patriarchal narratives to support justice for victim-survivors, such as targeted youth workshops on consent and digital harms, run in partnership with faith-based organizations and community leaders.

## Introduction

Fiji is a culturally diverse Pacific Island nation of less than one million people comprised primarily of Indigenous (iTaukei) Fijians, Indo-Fijians and other minority communities, such as Rotuman, Chinese Fijian and Pacific Islander groups (Ministry of Women, Children & Social Protection, 2023). Fiji has one of the highest rates of violence against women globally (Alam, 2025), with approximately 64–72% of women reportedly experiencing physical, emotional or sexual violence in their lifetime (FWCC, 2021). Technology-facilitated sexual violence, whereby digital technologies are used to perpetrate sexually abusive, harassing, coercive and exploitative behaviors, is also an emerging concern (Flynn et al., 2026; Flynn et al., 2022; Quilty & Flynn, 2025), with recent reports finding that over one-fifth of Fijian women experience such harm (Harkin et al., 2025; Ragio, 2024).

Within Fiji, patriarchal norms and religious interpretations inform societal responses to gender-based harm. Social norms and decision-making are shaped by traditional communal values, kinship systems, faith (predominantly Christianity, Hinduism and Islam), and respect for hierarchy (Amin et al., 2024; Clark, 2008; Saliya, 2024). These spiritual frameworks intersect with cultural ideals of modesty, family honor, and gender roles, historically positioning women as caregivers and men as protectors (Saliya, 2024). This includes patriarchal ideologies framing women as guardians of family and community honor, making them targets of scrutiny when that honor is perceived to be compromised, and conservative theological interpretations linking a woman’s worth to her sexual purity, implying that any perceived breach is a moral failing (George, 2025; Griffen, 2006). Although gender norms are evolving, patriarchal attitudes remain deeply embedded in Fiji, contributing to persistent gender inequalities such as limited female representation in leadership and the normalization of gender-based violence (Amin et al., 2024; Baker & Palmieri, 2024). These dynamics are compounded by institutional and social responses that reflect cultural biases toward victim-survivors, and to women generally, particularly those who hold positions in public life. Indeed, women who challenge traditional roles or assert themselves in leadership are often viewed as deviating from accepted norms, which can lead to social sanctioning and moral scrutiny (Saliya, 2024). This dynamic parallels with how victim-survivors of gender-based and sexual violence are treated, whereby women are frequently blamed for their own victimization, especially if they are perceived to have acted outside culturally prescribed boundaries.

Sexual and cultural taboos, strongly ingrained within traditional iTaukei and Indo-Fijian value systems, also contribute to moralistic victim-blaming and silence around sexual harm (Ireland et al., 2025; Naz, 2014). In Fiji, sex is often regarded as a private and shame-laden topic, particularly for women and young people, which severely restricts open dialog on sexuality and consent (O’Connor et al., 2019). This pervasive ‘sexual silence’ (Cammock et al., 2022) fosters misinformation and stigma, creating a social environment where candid conversations about sexual health are discouraged, with significant implications for those experiencing sexual violence, including emerging forms of sexual violence, such as image-based sexual abuse.

Image-based sexual abuse is a form of technology-facilitated sexual violence involving the non-consensual creation, capture, distribution, or threatened distribution of nude or sexual imagery (Flynn & Henry, 2021; Flynn et al., 2024; Henry et al., 2021). Such material may be self-produced but later misused, covertly obtained, or synthetically generated through AI and digital technologies, commonly referred to as “deepfakes” (Flynn et al., 2022, 2025a). A wide range of terms have been used to describe these behaviors, such as “non-consensual dissemination of intimate images” (Zvi et al., 2026), “non-consensual forwarding of sexts” (Maes et al., 2023) and, most contentiously, “revenge pornography” (see Henry et al., 2021 for a discussion on the harms of this term). In this paper, we adopt image-based sexual abuse, consistent with established scholarship (see e.g., Henry et al., 2021; McGlynn et al., 2021; McGlynn & Rackley, 2017), as it provides a comprehensive, victim-centered conceptualization of these abusive acts.

### Exploring Image-Based Sexual Abuse and Victim-Blaming Attitudes

Image-based sexual abuse is a serious and increasingly prevalent form of technology-facilitated sexual violence (Patel & Roesch, 2022; Powell et al., 2022, 2024), including in the Indo-Pacific, and Fiji specifically (Flynn et al., 2026; Harkin et al., 2025; Quilty & Flynn, 2025). An emerging body of research has begun to unpack attitudes toward image-based sexual abuse, including those that minimize the harms and place blame on victim-survivors (Bothamley & Tully, 2018; Flynn et al., 2023a, 2025b; Scott & Gavin, 2018). Much of this has been conducted in high- and middle-income countries, such as Australia, the United Kingdom and the USA, where gendered differences have been found in blame attribution, for example, men being more likely than women to blame victim-survivors and to perceive the abuse as less serious (Bothamley & Tully, 2018; Flynn et al., 2023a, 2025b; Scott & Gavin, 2018). Other research has found that greater blame is attributed to victim-survivors when images are consensually taken, but non-consensually shared, or when the nudity is perceived as more explicit (Crawford & Popp, 2003; Endendijk et al., 2020; McKinlay & Lavis, 2020; Pacilli et al., 2024; Pina et al., 2021; Zvi & Shechory-Bitton, 2020). Sexual double standards have been found to further exacerbate victim-blame, particularly among young people, with girls being judged more harshly than boys (Lippman & Campbell, 2014; Ricciardelli & Adorjan, 2019; Ringrose et al., 2013). This reflects a broader societal tendency to judge women based on perceived morality, reinforcing harmful stereotypes.

Emerging Pacific-focused research emphasizes that technology-facilitated sexual violence often intersects with cultural norms, relational dynamics, and geographic isolation, creating distinct vulnerabilities and gendered double standards (Flynn et al., 2026; Harkin et al., 2025). For example, coercive sharing of intimate images within extended family networks or community-based shaming practices reflects sociocultural dimensions not typically captured in Western frameworks. These intersecting cultural and structural factors not only exacerbate the harms of image-based sexual abuse, as victim-survivors face reputational damage and social exclusion, they also shape community attitudes toward victim-survivors. One manifestation of these attitudes is victim-blaming, which operates as a significant barrier to disclosure and support. Understanding this dynamic within Fiji’s sociocultural context is essential for situating local experiences within broader global patterns.

### Victim-Blaming in Fiji

Research with image-based sexual abuse victim-survivors indicates that victim-blaming attitudes can act as barriers to disclosure and help-seeking (Bates, 2017; McGlynn et al., 2021). This reflects research examining attitudes toward rape myths (false beliefs that excuse perpetrators and shift blame onto victim-survivors) and sexual violence victim-survivors more broadly. Informed by a nationally representative survey (n = 1500) in Fiji, Amin et al. (2020) argue that individuals who endorse traditional gender roles are more likely to accept rape myths, particularly when victim-survivors are perceived to have violated normative expectations of femininity. In contexts like Fiji, where cultural norms around gender and morality are strongly enforced, these attitudes are especially influential and operate as a mechanism of social control, limiting women’s access to justice.

In Fiji, no research has examined attitudes toward victim-survivors of image-based sexual abuse, however several studies have explored victim-blaming and domestic violence. Amin et al. (2024), for example, explore how cultural and religious frameworks in Fiji both resist and contribute to victim-blaming attitudes toward victim-survivors of domestic violence. They argue that traditional Fijian cultural values emphasize female submissiveness, the preservation of family unity, and maintaining social order for the benefit of the collective, which can result in victim-survivors being blamed for disrupting social harmony when they report abuse or seek help. Furthermore, women may be perceived as responsible for provoking violence through their behavior or failure to conform to expected gender roles, with this cultural framing reinforcing silence and under-reporting (Amin et al., 2024).

Religious institutions play a dual role in shaping community responses to victim-survivors. While some religious leaders promote care and compassion, others reinforce patriarchal interpretations that encourage women to endure suffering, particularly for the sake of marital sanctity (Aimen et al., 2024). Newland (2016) examined how customary practices and sociocultural expectations contribute to the normalization of violence and the marginalization of victim-survivors in Fiji, for example, through the traditional ritual of *bulubulu,* a customary iTaukei ritual of atonement and reconciliation traditionally used to restore harmony after disputes or wrongdoing. Historically, *bulubulu* aimed to break cycles of vengeance and maintain social cohesion, with the apology usually directed to the senior male member of the victim-survivors’ family, rather than the victim-survivor themselves (Clark, 2008; Ravuvu, 1983). While intended to repair harm, its use in cases of domestic and sexual violence has been criticized for prioritizing male relationships and community cohesion over justice and reinforcing the notion that women must bear the burden of maintaining social harmony, even when they are harmed (Newland, 2016).

Newland (2016) further argues that in Fiji, women are culturally positioned as caregivers and moral anchors of the family, and any disruption, such as speaking out against abuse, is seen as a threat to that communal stability. This dynamic fosters victim-blaming attitudes, where women are held responsible for the violence they experience; either for failing to conform or for challenging male authority. Saliya (2024) further explores this in relation to religious and ritual practices, such as the exclusion of women from kava ceremonies (used to mark important social or cultural occasions), which she argues reinforces male dominance and contributes to a public narrative that associates female visibility with impropriety. Saliya (2024) suggests that religion can be used as a tool of social control, legitimizing gender hierarchies and discouraging women from seeking justice or leadership. In this context, victim-blaming becomes a mechanism of cultural regulation, where women are held responsible not only for their own suffering, but also for disrupting social harmony.

Drawing on qualitative interviews with community elders and chiefs, Stamatakis (2024) normalized within familial and communal structures in Fiji. He highlights how gender norms and moral expectations reinforce a culture of silence and compliance, where victim-survivors are discouraged from seeking formal justice or support, and that attributions of blame occur when they do. He found that law enforcement responses are also shaped by these views; a finding that resonates in research outside Fiji (Flynn et al., 2023b; Henry et al., 2018). In the Fijian context, these systemic failures intersect with deeply rooted religious and cultural norms around sex, which shape attitudes toward sexuality and victimhood, including influencing perceptions of sexual behavior, both consensual and non-consensual (Varani-Norton, 2014).

### Sexual Taboos

Naz (2014) contends that in some contexts, particularly in rural locations and among certain ethnicities in Fiji, talking about sex is considered taboo, dating is discouraged, and sexual activity is expected only within marriage. In their study, Mitchell and Bennett (2019) highlight how young iTaukei women in Suva navigate sexual risk within a complex web of cultural expectations, personal desires, and fear of social judgment. They found that young women expressed concern about being labeled promiscuous, damaging their family’s reputation, or experiencing emotional harm in relationships, and these intersecting pressures reflected the broader gender norms and conservative values that shape how sexuality is understood and regulated in Fijian society (Mitchell & Bennett, 2019). These findings are directly relevant to how young people’s attitudes toward victimization are shaped in Fiji, whereby the same fears influencing young women’s sexual decision-making also contribute to negative attitudes toward victimization, reinforcing silence and feelings of shame, which can deter victim-survivors from seeking support after experiencing sexual violence (O’Connor et al., 2019).

Despite the growing prevalence of image-based sexual abuse, and studies exploring victim-blame and sexual taboo narratives in Fiji, scholarly engagement with the influence of cultural, religious and moral frameworks on perceptions of victimhood and culpability remain limited. This paper seeks to contribute knowledge to this area by drawing on the voices of young Fijians aged 18–24 years.

### The Present Study

Examining young people’s attitudes within the intersecting cultural, religious, and moral expectations of Fiji is critical to understanding the mechanisms through which these norms perpetuate victim-blaming and stigma toward victim-survivors, and hinder supportive responses to sexual harm. This paper presents a reflexive thematic analysis of data collected in focus groups conducted with 30 young people aged 18–24 years across three Fijian cities (Suva, Labasa and Lautoka) to understand: (1) what attitudes young Fijians hold toward victim-survivors of image-based sexual abuse; (2) what factors influence these attitudes; and (3) what impacts these attitudes have for victim-survivors of image-based sexual abuse. These questions aim to illuminate the underexplored intersection of cultural norms, gendered expectations and digital harms in Fiji, providing a culturally grounded contribution to global scholarship on technology-facilitated sexual violence. Young people’s attitudes toward image-based sexual abuse are further explored using the illustrative case study of former Fijian Minister for Women, Children and Social Protection, Lynda Tabuya, who had a private sexual video disseminated online without her consent and was consequently dismissed from her Ministerial position. In doing so, this paper explores how gender and cultural norms shape young people’s perceptions of victimization status and worthiness in this specific case, but also more broadly of their peers, friends and other women victim-survivors.

In exploring the cultural, social and religious contexts operating in Fiji, and what it means to be a victim-survivor of sexual harm which transgresses populist views around morality and purity, this paper seeks to contribute to the growing body of literature that links patriarchal norms to the perpetuation of victim-blaming and underreporting of gender-based violence in Fiji (Newland, 2016; Stamatakis, 2024). The paper begins by outlining the research methodology. It then presents the results, followed by a discussion of the key findings, future directions for researchers, policy and practice, and the study’s limitations.

## Method

### Participants and Procedure

In June 2025, six in-person focus groups were held with 30 Fijians aged 18–24 years in Suva, Labasa and Lautoka to gain deep, context-specific insights into young people’s understandings and experiences of technology-facilitated gender-based violence, including their attitudes toward victim-blaming and image-based sexual abuse. This was part of a larger study that sought to understand: (1) the nature and impacts of technology-facilitated gender-based violence experienced by young people in Fiji; (2) attitudes toward technology-facilitated gender-based violence victimization and perpetration held by young people in Fiji; and (3) the ways in which technology is used—positively and negatively—by young people in Fiji. We focused on young people aged 18–24 years because this demographic is among the most active users of digital technologies and social media in Fiji, which are key platforms where image-based sexual abuse occurs (Harkin et al., 2025; Quilty & Flynn, 2025). This age group also represents a transitional cohort; young adults who are deeply embedded in Fiji’s cultural and religious norms, yet navigating new digital environments. Their perspectives are critical for understanding how traditional frameworks of morality, purity, and victim-blame intersect with emerging online risks.

In this paper, we draw upon a specific set of questions (detailed below) and discussions from the focus groups to explore young people’s attitudes toward one specific form of technology-facilitated gender-based violence—image-based sexual abuse—using the high-profile victimization experience of Fijian Member of Parliament, Lynda Tabuya, to generate discussion.

A purposive sampling approach was used for the focus groups in which participants were selected based on their age (18–24 years). Participants were recruited using a mix of social media promotions on the research team’s professional social media profiles and networks; community outreach, such as posters placed on university campuses across the three focus locations in Fiji; and snowball sampling among participants. Anyone who expressed interest in participating completed a demographic questionnaire to ensure eligibility in age range, and to encourage diversity among demographics, where possible.

Recruiting young people in Fiji presents unique challenges, including geographical dispersion, limited transport infrastructure and cultural sensitivities (James, 2023). These factors, combined with the hard-to-reach nature of the cohort, constrained participation despite extensive engagement efforts. Overall, 103 individuals expressed interest in participating; 13 were unavailable during the research period, and 20 were ineligible due to falling outside the age range. The remaining 70 people were invited to participate, and ultimately 30 attended focus groups. Non-attendance was primarily due to non-response, changes in willingness to participate, or logistical barriers.

While this cohort represents less than half of those invited, the achieved sample size was sufficient to reach data saturation. Empirical evidence indicates that saturation in focus group studies typically occurs within three to six groups when participants share similar characteristics and experiences (Guest et al., 2017; Hennink & Kaiser, 2022). Our study included multiple focus groups with participants drawn from three diverse locations. Choosing Suva, Labasa and Lautoka allowed the study to capture Fiji’s urban, rural, and industrial contexts, highlighting variations in cultural norms, digital access, and support systems. This provided variation in perspectives, while still meeting the conditions under which saturation is commonly observed. This approach aligned with established guidance that saturation depends more on the richness and diversity of data than on absolute numbers (Hennink & Kaiser, 2022). The demographics for the focus groups are provided in Table 1. All participants received a phone credit voucher worth $30 (FJD) to acknowledge their time and contribution.

**Table 1:** Socio-demographic characteristics of the sample (n = 30)

Characteristic	Number	%
*Age* (*years*)		
18–20	13	43.3
21–24	17	56.6
*Gender*		
Female	17	56.6
Male	13	43.3
*Sexual orientation*		
Straight	23	76.6
Same sex attracted, bisexual	3	10.0
Prefer not to say	4	13.3
*Town/city*		
Labasa	2	6.66
Lautoka	8	26.6
Suva	10	33.3
Elsewhere in Fiji	10	33.3
*Ethnicity*		
iTaukei	15	50.0
Indo-Fijian	11	36.6
Fijian of Chinese descent	2	6.6
Rotuman	2	6.6
*Faith group*		
Catholic	2	6.6
Christian	19	63.3
Hindu	3	10.0
Muslim	4	13.3
Prefer not to say	2	6.6

### Ethical Considerations

All participants received clear information about the study’s objectives, procedures, and their rights prior to participation, with informed consent obtained both verbally and in writing, in line with international research guidelines (WHO, 2016). Focus groups, composed of participants diverse in gender, age, and ethnicity, were organized by geographic location and conducted using trauma-informed, safety-conscious and culturally responsive practices appropriate to the sensitive nature of technology-facilitated gender-based violence. Confidentiality was assured through anonymised transcripts and secure data storage, and each session was co-facilitated by three researchers trained in trauma-informed and qualitative methods, who also provided structured debriefings and information on local counseling services. Guided by Pacific ethical principles of relational engagement, respect and reciprocity (Vaioleti, 2016), facilitators fluent in English, iTaukei, and Hindi employed culturally sensitive and neutral questioning techniques to reduce power imbalances and foster trust, including accommodating customary communication preferences, such as initial use of iTaukei in one focus group, before participants transitioned comfortably to English.

### Measures

#### Focus Group Questions

The focus group questions were divided into four sections: positive uses of technology (e.g., “What are the key benefits of technology use for young people?”); perceptions of technology-facilitated gender-based violence (e.g., “What types of technology-facilitated gender-based violence have you heard of?”; “Why do you think people engage in these behaviors?”); harms and impacts (“What are some of the ways image-based sexual abuse can harm people?”, “Are victim-survivors responsible when they experience image-based sexual abuse?”); and scenario questions, in which we provided case studies of hypothetical and real instances of image-based sexual abuse and asked questions around the behavior, perceived motivations, harms, bystander intervention, and legal and other responses. The use of scenario-based questions is common in qualitative research to elicit nuanced responses and stimulate discussion on complex social issues (Braun & Clarke, 2021a; Flynn et al., 2025b). The scenarios were designed to align closely with the study’s research questions by illustrating real world examples of image-based sexual abuse relevant to the Fijian context.

**Fig. 1:**
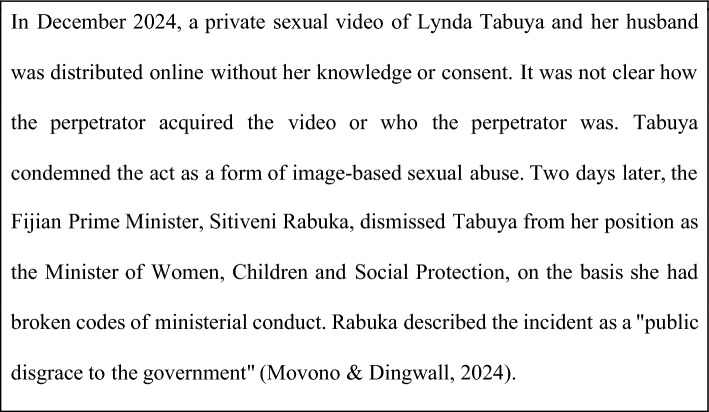
Lynda Tabuya case study scenario

The case study of Fijian Member of Parliament, Lynda Tabuya (see Fig. 1 below) was selected because it represents a recent, widely recognized and culturally salient instance of image-based sexual abuse in Fiji, thereby enhancing ecological validity and ensuring that discussions were grounded in participants’ lived realities (Henry et al., 2020). While the case study discussed involved the victimization experiences of an older (prominent) woman, our aim was not to replicate her experience, but to explore attitudes among those most likely to encounter image-based sexual abuse in their own lives or peer networks. This approach aligns with evidence that young people in Fiji face unique challenges related to sexuality, gender norms, and digital intimacy, making them a priority population for prevention and policy interventions (Ali-Traill, 2023; O’Connor et al., 2018). To achieve this, we provided a brief outline of the case study to participants (all participants were aware of the case) and asked a series of follow-up questions (e.g., ‘What are your thoughts around the video being shared?’; ‘who is responsible for the video being shared—is it the person who leaked the video or Tabuya?’; ‘would male ministers be held to the same standard?’).

The focus groups lasted between 74 and 180 min, with an average duration of 110 min.

### Data Analysis

The focus groups were audio-recorded, transcribed (with identifying information removed) and analyzed using reflexive thematic analysis as outlined by Braun and Clarke (2019, 2021a, 2021b), which treats theme development as an interpretive, researcher-generated process rather than a purely descriptive or consensus-driven exercise. We selected this approach for its flexibility in identifying and interpreting patterns within qualitative data, its combability with an inductive and interpretivist orientation, and its emphasis on moving beyond surface description toward patterned meaning and a coherent analytic narrative. The coding scheme was informed by this paper’s focus on the Tabuya case study and grounded in literature on victim-blaming and gender-based violence, ensuring the analysis captured both culturally specific and theoretically relevant dimensions.

The analytic process proceeded inductively, attending to both explicit and underlying meanings. The process was recursive and iterative, with the lead and the second author immersing themselves in the transcripts to become familiar with the data, then generating initial codes that captured nuanced expressions of participants’ attitudes toward victimhood, purity, and blame. Initial coding identified categories such as victim-blame, gender blame, taboo, and religion. These codes were then synthesized into broader interpretive themes explaining how cultural and moral frameworks shape young people’s attitudes toward image-based sexual abuse (e.g., attitudes of victim-blame, responsibilizing the victim-survivor and absolving the perpetrator). Themes were refined through team discussions to ensure coherence and depth, with attention to contradictions and variations across groups. Coding and theme development were managed using Dovetail. To enhance analytic reflexivity, transparency of decision-making and critical dialog (Braun & Clarke, 2021a), the lead and the second author independently coded the transcripts, and met together to discuss their reflections. As part of these discussions, the two lead authors engaged in reflexive practices, including discussing how their own personal and professional backgrounds may influence their assumptions and expectations of young Fijians, and how these might have shaped coding decisions. We used these reflexive practices to contextualize our interpretations, acknowledge our subjective analytic role and ensure the themes presented were well-evidenced within the dataset.

Participants’ reflections are identified by a focus group number (e.g., FG1, FG2), location (e.g., Suva, Labasa, Lautoka), and gender of the participant (e.g., Male, Female). The number of participant quotes presented within each theme reflects the relative prominence and richness of data, rather than an attempt to equalize evidentiary weight. Some themes were highly saturated, warranting multiple illustrative examples, whereas others appeared less frequently and were expressed in more generalized terms. This variation is consistent with qualitative reporting standards, which prioritize authentic representation of patterns in the data over numerical parity (Braun & Clarke, 2006). Our aim was to maintain analytical coherence and avoid artificially inflating less prominent themes, while ensuring transparency through the inclusion of representative quotes.

## Results

### Attitudes of Victim-Blame, Responsibilizing the Victim-Survivor and Absolving the Perpetrator

In discussing image-based sexual abuse, the focus groups revealed deeply entrenched patterns of victim-blaming attitudes held by participants. These attitudes were often aligned with negative perceptions around sexual expression, which obscured the non-consensual nature of the abuse, resulting in responsibility being attributed to the victim-survivor and reduced culpability on the perpetrator. As these participants reflected: “They [victim-survivors] bring it upon themselves” (FG1 Suva Female) and “When you don’t do anything bad, nothing bad is spread about you” (FG1 Labasa Male). This reasoning illustrates how moral discourses intersect with victim-blaming attitudes, positioning responsibility with the individual rather than the perpetrator, a pattern consistent with gendered norms documented in Pacific contexts (Power, 2023). Such statements reinforce the idea that image-based sexual abuse is avoidable through “good” conduct, reflecting broader cultural narratives of morality and respectability.

Victim-survivors were further criticized for their choices, with participants questioning why individuals would share sexual images of themselves or allow other people to take sexual images of them. FG1 Suva Female, for example, stated, “I sometimes think that those people deserve it, because of how they—they carry themselves.” By framing image-based sexual abuse as a moral transgression rather than a breach of consent, this participant invokes a discourse of respectability that positions a victim-survivor’s worth in relation to perceived propriety and sexual conformity. Such views align with documented gendered norms that exist in Fiji where morality and reputation are central to social identity (Amin et al., 2024). By positioning harm as deserved, this statement illustrates how victim-blaming attitudes intersect with cultural expectations of behavior, reinforcing structural gender inequalities.

A common theme emerging across the focus groups was a belief that when image-based sexual abuse cases come to light, Fijians are overwhelmingly critical of victim-survivors, rather than perpetrators. As one participant observed:

When a nude gets sent around, people just focus on the victim, the person inside the video, [rather] than the person sending it. … There’s not much focus on the perpetrator. … They tear that victim down. (FG2 Suva Female)

Another participant similarly reflected that “they just throw shade at the victim. Because why would she be doing that in the first place?” (FG2 Suva Female). These views were particularly strong when the victim-survivor had initially consented to the image being taken or had shared it with a partner, who then non-consensually circulated the image. Several participants placed additional blame in these contexts, reflecting that people are warned to be careful with what they share online or through digital technologies, including with partners, so there is additional judgment applied:

Be careful what you post, you know, or share online. Do not share even to your-would-be-trusting partner, [as] you never know. Because you two might not stay together for the long run. You never know what’s your future, your tomorrow. So don’t even share. (FG1 Suva Female)

In emphasizing a focus on self- protection, this perspective further shifts responsibility for preventing image-based sexual abuse onto potential victim-survivors rather than perpetrators, reinforcing a discourse of personal accountability. This logic reflects broader moral narratives that prioritize individual responsibility over structural solutions, further highlighting the role of gender norms in influencing young people’s perspectives in Fiji.

In reflecting specifically on the Tabuya case study, participants expressed victim-blaming attitudes toward her, criticizing her decision for having been involved in the making of a sexual video in the first place, even though this was a consensual act with her partner. FG1 Lautoka Male stated, “I feel that she’s accountable.” Another participant similarly observed, “even though she’s the victim of cyberbullying, and without her consent that picture was leaked, you think, what kind of lady would do that? Like she’s a grown ass woman” (FG1 Suva Female). Other participants reflected on how this perception toward Tabuya was commonly held by people across Fiji. As one participant explained, “in terms of Fiji social norms, very much everyone will agree that she was at fault” (FG1 Lautoka Female).

This moral scrutiny explicitly links victim-blaming to cultural consensus, underscoring how image-based sexual abuse is interpreted through collective moral frameworks in Fiji that position women as responsible for maintaining respectability. The invocation of “social norms” highlights the normative power of community expectations in shaping young people’s attitudes toward sexual harm. Further, by framing blame as socially sanctioned, this perspective illustrates how cultural norms legitimize punitive responses to perceived transgressions, reinforcing structural gender hierarchies. This moral scrutiny is echoed in Johnson et al.’s (2019) study on intimate partner violence in Fiji, which found that female victim-survivors who were perceived as violating sexual norms received less empathy, while perpetrators of violence against these women were judged more leniently. This dynamic was particularly evident in contexts where women’s behavior was closely scrutinized against moral expectations.

Our focus group discussions similarly revealed a focus on the victim-survivor’s behavior (e.g., why the video was taken), rather than the perpetrator’s violation of consent (e.g., sharing the video without permission or approval). Reflecting again on the Tabuya case study, one participant explained:

What she did was wrong, but, um, it was wrong for the person to have shared the video. … But then I think that there will still have to be some repercussions against what she has done. Otherwise, it will just start a culture of doing something wrong like that. (FG1 Suva Male)

In this reflection, the “something wrong” Tabuya may “start a culture of doing,” referred to the making of a sexual video between two consenting parties (her and her husband). The removal of accountability from the perpetrator who disseminated the video was further evident in other comments where responsibility was placed on Tabuya because she was involved in creating the video, and had failed to prevent it from being shared:

Why should you make a video when you can’t keep it, if you can’t handle it safely. Why do you make it? It was private. It was safe apparently. Somebody either hacked it or released it. I believe if she took the necessary precautions, it wouldn’t be out like that. (FG2 Lautoka Male)

Another participant similarly commented, “you shouldn’t make it in the first place or have it in the first place, because, like, prevention is better than cure” (FG1 Suva Male).

Blaming or focusing on the victim-survivor in this way shifts attention away from the actions and accountability of the perpetrator. Indeed, the narratives evident in these comments implies that Tabuya’s behavior is the cause of the abuse, rather than the deliberate and harmful choices made by the perpetrator. As a result, it normalizes the abuse, discourages reporting, and reinforces a culture where perpetrators are not held responsible for their actions. This has the potential consequences of undermining justice and perpetuating cycles of harm not only in the case study of Tabuya, but also for future victim-survivors of image-based sexual abuse.

### A Culture of Silence

One of the key themes emerging across the focus groups, which was linked to attitudes that placed blame upon victim-survivors, was the taboo nature of sex, which creates a barrier to addressing abuse and to supporting or empathizing with victim-survivors. Highlighting how cultural taboos around sexuality create conditions for judgment rather than empathy, one participant explained:

You know, being Fiji, it’s [sex], it’s quite a taboo topic, right? So, no one’s really open to discuss it. … Culturally, you’re not allowed to talk about sexual things or discuss what you’re going through. … So, when it [image-based sexual abuse] happens, you can judge [the victim-survivor]. (FG1 Suva Female)

This comment illustrates how silence around sexual topics reinforces moralistic responses to image-based sexual abuse, positioning victim-survivors as blameworthy for transgressing norms of propriety. Such dynamics are consistent with sexual shame and moral regulation in Pacific societies, where cultural prohibitions on discussing sex amplify stigma (Naz, 2014). The cultural taboos and shame around sex were also found to impact on the help-seeking behaviors of those who experience sexual victimization. In describing a friend’s experience of image-based sexual abuse, this participant’s comments highlight how victim-survivors themselves internalize cultural norms of silence, instead of seeking help. He noted, “it was a level of taboo that even she didn’t want to speak to, outside of us [her friends], which I understand 100%. I accept it” (FG2 Suva Male). This reluctance to disclose beyond trusted peers further reflects the pervasive fear of social judgment and reputational harm, which research identifies as a key barrier to reporting sexual harms (Wieberneit et al., 2024). By normalizing secrecy, these norms perpetuate cycles of victim-blaming, silence and isolation.

The focus group discussions revealed that in Fiji, young people, particularly teenagers, are hesitant to reach out to parents for support about image-based sexual abuse, fearing both judgment, blame and punishment, such as their phones being taken away:

For Fijians, I don’t think we open up to our parents very much, cause even if we were to say it, they’ll always look at the negative side of it, they won’t look at the positive side. They will just blame us for it. Like what we were doing? Why were we engaging in it? (FG2 Lautoka Female)

These views provide an insight into some of the intergenerational tensions in responses to image-based sexual abuse. Participants commonly described a cultural dynamic where parents tend to focus on the potential shame or reputational aspects of the behavior, rather than understanding the full context, which resulted in a reluctance to seek help to avoid any stigma. In reflecting on her friend’s experience of image-based sexual abuse by a former partner, one participant reflected on the ethnic and cultural variations in moral expectations, describing how her friend had reached out to a supported family member for help, and she was not only blamed for the non-consensual sharing of the images by her ex-partner, but her reputation was criticized, and she was alienated from the family. She claimed, ‘you know how Indo-Fijian, like, households are, … so they were really upset with her. They were like, “We don’t want to see you now”. There was a lot of bad things that happened’ (Lautoka FG2 Female). This description shows how families themselves can act as enforcers of rigid norms around sexuality and reputation, amplifying responses of victim-blaming and social judgment to create a norm that deters disclosure.

Linked to these responses, the focus groups also revealed tensions between Fiji’s religious and cultural identity, technology, and image-based sexual abuse victimization, creating complex challenges for victim-survivors. Indeed, participants pointed to the conflicts some of them felt between their faith and their desire to engage with modern technology, such as social media. As one participant reflected:

There’s a big conflict on our ethics side, especially as Christians. … So, if we post online, an example is, especially university students going clubbing and having fun outside going to church, then next week, on Sunday … [we face] the judgmental community. And you get that reputation that will hold on to you, because there’s a stigma to it. (Lautoka FG2 Female)

In particular, there was a common perception that certain religious and cultural communities place judgment on individuals who engage in behaviors that are perceived as morally or socially unacceptable, with participants noting that some faith-based counseling could be judgmental, rather than supportive, when dealing with issues like image-based sexual abuse or online harassment. As one participant explained, ‘that’s a common experience I’ve seen among Fijian culture, it’s just sort of, “you’re not supposed to be doing this [sex], … we will judge you on this group of sins”’ (Lautoka F1 Female). The tension identified by this participant reflects broader research on Christianity’s influence on gender and sexuality norms in the Pacific, where religious morality often amplifies reputational concerns and victim-blaming (Rallu, 2018; Trompf, 2023). Such stigma can lead to a reluctance to seek help or support, even in situations of crisis or distress. Connected to this, participants also described situations where victim-survivors turned to their families for assistance, yet concerns about preserving the family’s standing within religious and cultural networks led to a lack of support. One participant, recalling an incident of image-based sexual abuse, noted:

She was really suicidal and reached out for help. People were not willing to help her because it was that, you know, their reputation was at risk. … She shared, like, the situation with, a senior [family member]. She was like, ‘Can you guys go and, like, inform the police, that all of this has happened’, and they were like, ‘Oh, why should we go and do that? It’s like, you’ll get, like a really bad image in the community as well’. They said, ‘if the police come over, then, like everybody [will know], like neighbors especially, they’re really nosey’. (Lautoka FG2 Female)

This excerpt illustrates the collectivization of shame and the displacement of responsibility from the perpetrator to the victim-survivor and her family. The family’s reluctance to engage law enforcement reflects a moral economy of reputation, wherein the imperative to preserve communal standing is prioritized above the victim-survivor’s needs.

In another focus group, a participant reflected on how a situation of image-based sexual abuse involving a consensual sexual encounter that was non-consensually filmed and disseminated, ultimately resulted in sexual stigma and shame being directed toward both the victim-survivor and her family, but not the perpetrator:

This boy and a girl, they had sex, and then the girl didn’t know that the guy was filming her, and then he forwarded the video to his best friend. And then his friend posted the video and then like the girl, the whole family, they were all degraded. (FG1 Lautoka Female)

Here, the harm is extended beyond the individual to an entire familial network, signaling the operation of collective stigma. The language of ‘degradation’ situates image-based sexual abuse within a framework of communal morality, wherein sexual transgressions, real or perceived, are interpreted as violations of family honor, rather than breaches of consent. This reflects gendered moral hierarchies, positioning women as custodians of familial virtue and rendering their bodies symbolic capital within patriarchal systems. Meanwhile, the male perpetrator’s responsibility is erased, thereby sustaining structural inequalities. The intersection of digital technologies with these normative logics amplifies vulnerability, transforming a private act into a public one and intensifying the reputational harm beyond the individual to her entire family (McGlynn & Rackley, 2017). In this context, the cultural pressures may lead families to remain silent, prioritizing social standing over justice or healing, and leaving victim-survivors without the support they need.

### Gendered Double Standards and Moral Policing

Overwhelmingly, the focus groups revealed how victim-blaming and gendered double standards work together to create a particularly hostile environment for women experiencing image-based sexual abuse in Fiji through a form of moral policing. Participants reflected on a prevalent patriarchal mindset and culture that views men as ”superior,” leading to women being treated with limited authority and agency. As one participant observed, “in the Fijian tradition, males are always considered superior. So, the focus is always on them” (FG2 Suva Female). This statement foregrounds the structural gender hierarchy embedded in Fijian cultural norms, where male superiority is normalized and institutionalized. In this context, the “focus” on men operates paradoxically: while men dominate social attention and authority, they evade moral sanction, reinforcing a gendered asymmetry in blame attribution.

This hierarchy is further evident in how incidents are interpreted and responded to differently based on gender, with women held to higher ethical and behavioral standards compared to men, with their actions and choices being more harshly criticized and judged. As FG1 Suva Female reflected, “When women are judged it’s different, you know, even if they do like the same [thing]. … The criticism that the girl will receive, it’s much worse, because the expectation they just put on women is too much.” FG2 Suva Male likewise explained, “I think women are held to a different standard when it comes to issues of ethics. … They are expected—they’re held to a much higher standard than what our male counterparts would be held to.” These comments explicitly name the ethical asymmetry that structures gender relations, situating image-based sexual abuse within a broader moral economy that disproportionately regulates women’s conduct. In image-based sexual abuse cases, these expectations translate into intensified blame and reputational harm for women, while men’s transgressions are trivialized. Such findings echo global scholarship on image-based sexual abuse, which identifies gendered moral frameworks and moral policing as central to sustaining victim-blaming and impunity for perpetrators (Flynn et al., 2023a; McGlynn & Rackley, 2017).

The focus groups also revealed a strong tendency to blame and shame women for issues or problems, even when they were the victim-survivors. Participants attributed this to instances of image-based sexual abuse, and to women experiencing domestic violence. As FG1 Lautoka Male explained, “If a female would, for instance, show a nude picture of herself, people would assume that she’s a slut or something like that. But if a guy does it, it’s, ‘Oh, he’s self-confident of his body.’” Another participant reflecting on other forms of sexual and domestic violence also observed, “If her husband is abusing her, she’s also at fault because, you know, she is not keeping her husband happy” (FG1 Suva Female).

When asked why this gendered double standard exists, the dominant responses provided by participants were: “tradition” (FG2 Suva Female), “patriarchal values” (FG1 Suva Female), and “the old mindset” (FG3 Suva Female). Using an example of the differences between how men and women are viewed in business to explain these gendered norms, one participant continued:

Apart from Fiji, other countries, everyone has moved on from that mindset. If you go to [the] US or Singapore, those countries, you can see a woman actually working in very high positions. Compared to Fiji, if a woman is starting her business, they will talk her down. They’ll be, ‘Oh, you won’t be able to’. Whereas a man is starting a business, ‘yeah, he’s got it, he’s a man. He knows what to do in business’. So, that perspective hasn’t left the mindsets of people in Fiji. (FG3 Suva Female)

As illustrated in this reflection, the influence of patriarchal traditions in Fijian society identified across the focus groups was pervasive and complex, and was a key factor shaping attitudes toward gender roles and social interactions. It was also influential in how participants viewed perpetrators of image-based sexual abuse, compared to the victim-survivor: “like if you’re the person that’s sharing it, then you’re like more looked up to, because like you shared it” (FG1 Lautoka Male).

The ways in which gender bias manifests in attitudes toward women and victim-survivors was particularly highlighted in the Tabuya case study, where participants reflected that women politicians face more intense scrutiny and backlash compared to their male counterparts. The following observations are reflective of this view: “If the same situation like happened to a guy, like if he was a member of the parliament, he would still have had that seat” (FG3 Suva Female), and “there is the double standard. … Like male ministers … are doing things that are questionable, but still, they can do whatever they want” (FG1 Suva Male).

Across these comments, participants were articulating a gendered moral regime, whereby male dominance and female respectability were evident not merely as cultural ideals, but active mechanisms of social control, shaping how harm was interpreted and responsibility assigned. Rather than focusing on the violation of Tabuya’s privacy or on the actions of the perpetrator, participants reflected on how the public discourse (and often their own views) centered on her failing as a woman, a wife, and a mother. FG1 Labasa Male stated, “She’s a married woman and she’s doing things that she shouldn’t be doing.” FG1 Suva Female likewise claimed, “She’s almost like a grandmother now. She has children, they are older and married. So why is she trying to be like us and like young people, wanting to, sharing something like that. … Why is she sharing that?”

Participants also strongly criticized Tabuya for breaching her role model status as a woman in power. FG1 Labasa Male stated “doing that [a sexual video] wasn’t showing good leadership.” FG1 Suva Female offered similar views, claiming “She’s ruined the country’s reputation. We thought she’s like the first woman, because she’s one of the women like we were—even we had voted for her. She’s our hope. She was like a role model for young girls.”

Even where participants recognized the gendered double standards that were being applied to Tabuya, there was still an attribution of blame placed on her for having been in a situation where a sexual video could be shared. For example, one participant claimed, “like even if a male politician, something comes out about them, like they won’t really criticize because it’s a man. But for girls, it’s different, you know. … She actually spoiled our opportunity for girls” (FG1 Suva Female). Another commented that:

Now people will not see her as how they used to see her before, like as Lynda Tabuya. They will see her as someone who sent a nude video around. Like, if we see her now, it’s like, “Oh, that’s her. She sent the video.” Instead of seeing her how we used to see her before. (FG2 Suva Female)

These remarks convey hostility toward Tabuya, driven by the notion that she transgressed the normative expectation of a “pure, non-sexual” female identity, thereby exemplifying moral policing. Women in politics are often subjected to this additional critique, where their actions, speech, and personal lives are scrutinized more intensely than those of their male counterparts (Chattier, 2015; George, 2012). This scrutiny reflects broader societal expectations that women in public life must uphold idealized standards of femininity and virtue. Any deviation, such as assertiveness, political ambition, or involvement in controversy can provoke public shaming and de-legitimization. This is certainly reflected in the focus group participants’ responses to the Tabuya case study, in which her victimization and political role was framed through a gendered lens that demanded conformity to traditional roles of modesty, morality, and familial responsibility; something she was perceived as failing to do because she was a victim-survivor of image-based sexual abuse.

## Discussion

The research findings indicate that victim-blaming attitudes held by young Fijians toward victim-survivors of image-based sexual abuse in our study are shaped by entrenched patriarchal norms and moralistic frameworks that police victim-survivors, primarily women, based on perceived transgressions from cultural norms, rather than judging perpetrators, primarily men, for their violations of consent. When women experience violence, discrimination, or reputational harm, they are frequently blamed for violating social norms or “inviting” mistreatment (Amin et al., 2024; Johnson et al., 2019). In this sense, the same logic that questions a woman’s suitability for leadership, based on perceived moral failings (George, 2012), also underpins societal responses to image-based sexual abuse. Women are expected to maintain social harmony, and when harm occurs, they are often seen as responsible for having disrupted that balance.

George (2012, p. XLIII) documents how women’s entry into public life in Fiji has often been met with skepticism and resistance, rooted in patriarchal norms that associate leadership and authority with masculinity, whereby “women’s interests are separate to those of men”. Public attitudes toward such women are influenced by entrenched cultural expectations that prioritize women’s roles as caregivers and moral guardians of the family. These roles are often seen as incompatible with political engagement. Therefore, when women in public life deviate from these norms, including in the case of Tabuya (e.g., filming sexual acts with her partner), they are subject to public shaming; something George (2012, pp. CXII-CXIII) explored in relation to Adi Finau Tabakaucoro, a former civil servant and cabinet minister, who was publicly criticized for the outspoken ways in which she advocated for indigenous women’s rights.

Across the focus groups, our participants expressed attitudes that often placed responsibility onto victim-survivors of image-based sexual abuse, reflecting deeply embedded gendered norms around moral and cultural taboos that have been documented across Pacific societies (Griffen, 2006). Within these discussions, victim-survivors were judged not (primarily) for the abuse they endured, but for their perceived moral failings, such as engaging in sexual expression. This framing aligns with broader patriarchal norms in Fiji that construct women’s social value around modesty, sexual purity, family honor and conformity to traditional gender roles (Amin et al., 2020). In the context of image-based sexual abuse, when victim-survivors are perceived to have violated these norms, even involuntarily, they face social ostracism and blame, including, in the examples provided by our participants, by their own families, and religious communities.

Religious narratives further entrench these attitudes by promoting conservative gender roles and linking a woman’s worth to sexual purity. The findings informed by participants in our study suggest these narratives may extend to the help available for victim-survivors, including from their family, where fear of reputational damage can result in a failure to support those experiencing sexual harm or a deliberate focus on silencing victimization. As the focus group data suggests, these cultural logics not only stigmatize victim-survivors, but they diminish empathy for them and ultimately shift accountability away from perpetrators; an outcome clearly demonstrated by responses to the Tabuya case study, in which there was minimal discussion of the perpetrator, and instead, a dominant focus on Tabuya’s (perceived) actions in breaching her position as a woman, mother, wife and role model. While similar behaviors by men were normalized or overlooked by participants, Tabuya’s perceived transgression attracted disproportionate scrutiny, exemplifying the gendered double standards of moral regulation (see also Henry & Powell, 2016; McGlynn & Rackley, 2017).

Commentary on Tabuya’s engagement in sexual activities as an older woman were also evident in participant narratives, suggesting that among our participants, ageism may operate alongside gendered norms as a cultural determinant of victim-blaming attitudes. This intersection of age and gender reinforces moral discourses that position older women’s sexuality as transgressive, amplifying stigma and intensifying reputation harm in ways that compound patriarchal control (Jen, 2017).

Importantly, while the Tabuya case was prominent in our discussions, the attitudes held and critiques directed toward Tabuya’s experience were not dissimilar from those our participants discussed involving their peers and friends, suggesting that the scrutiny directed at Tabuya reflects a broader tendency to impose moral and behavioral standards on women generally. This framing aligns with Pacific scholarship on gender and sexuality, which underscores how women’s bodies and reputations are constructed as symbolic capital within patriarchal systems (Griffin, 2025; Kelly-Hanku et al., 2023). In this context, breaches of privacy, such as image-based sexual abuse, are reframed as evidence of personal irresponsibility, rather than as acts of perpetrator wrongdoing. The Tabuya case thus served as a cultural touchstone for participants in our study to articulate normative expectations of female respectability. By situating the Tabuya case within this broader moral economy, we see how the participants’ attitudes toward image-based sexual abuse are shaped by structural gender hierarchies that amplify women’s vulnerability and reduce accountability for perpetrators. This underscores the need for interventions that challenge these normative frameworks, moving beyond individual blame to address systemic inequalities embedded in both cultural and digital spheres.

A striking feature of the data was the coexistence of conflicting views. While some participants acknowledged perpetrator responsibility for image-based sexual abuse, others shifted blame toward victim-survivors, often invoking notions of personal accountability and caution. These tensions reflect the complexity of moral reasoning among the participants, where global discourses on consent intersect with local norms around gender, reputation, and respectability. Adding to this complexity, participants were frequently critical of their parents’ responses to image-based sexual abuse, describing them as harsh, judgmental, and rooted in victim-blaming. Several participants noted that parents framed image-based sexual abuse in ways that shamed rather than empathized with victim-survivors, yet young people simultaneously echoed these same narratives in their own reasoning, including describing image-based sexual abuse being the result of a victim-survivor’s bad choices. This suggests that while they reject the tone of parental responses, they reproduce the underlying norms. This pattern illustrates norm diffusion across generations, where gendered expectations and moral discourses persist, even as young people position themselves as more progressive than their elders.

These contradictions between acknowledging harm and reinforcing blame, and between critiquing parental attitudes while replicating them, further demonstrate the need for age-appropriate and culturally grounded interventions that address structural gender norms and intergenerational transmission of values. Such interventions must move beyond individual behavior change to challenge the broader moral frameworks that normalize victim-blaming in both public and private spheres (Flynn et al., 2025b).

### Future Directions and Study Limitations

The findings of this study contribute to a broader narrative challenging the hostile environment that patriarchal and moralistic norms can create for victim-survivors of sexual and gender-based violence (Amin et al., 2024; Johnson et al., 2019; Stamatakis, 2024). Our findings suggest that addressing image-based sexual abuse in Fiji requires interventions that confront these cultural norms, promote consent, and reframe public discourse around victim-survivor dignity and rights. Discussions of the Tabuya case highlight the importance of improved education and awareness of the harms of image-based sexual abuse which extend beyond legal reform. Indeed, participants recognized the abuse was a form of harm, and a criminal offense, but still placed blame and responsibility onto Tabuya.

Evidence from prevention frameworks highlights that early, age-appropriate consent education fosters respect for boundaries and challenges harmful gender norms (Basile et al., 2016; UNICEF, 2020). One way to achieve this in the Fijian context may be to explore changes to sex education, and improving knowledge of sexual boundaries, privacy, and the consequences of non-consensual image-sharing from an early age. This education could address cultural taboos by integrating modules on consent, online safety and respectful relationships. Education could also focus on the emotional and social impacts of image-based sexual abuse and the barriers to reporting, especially for women and girls who are disproportionately affected by blame. In Fiji, the potential to combine this with faith-based engagement is key. While participants identified tensions between their religious and social lives, developing youth-targeted workshops co-facilitated by educators and religious leaders could provide a mechanism for religious leaders to leverage their influence to challenge victim-blaming attitudes and promote respectful digital ethics.

Future research on image-based sexual abuse among young people in Fiji must also adopt culturally grounded and intersectional approaches to address the complexity of this growing issue. Our study suggests that victim-blaming remains a pervasive barrier to disclosure and justice for victim-survivors of image-based sexual abuse, yet more research is required to understand how to challenge these attitudes within Fiji’s unique sociocultural context. Drawing on our findings which suggest some sense of pervasiveness of forms of technology-facilitated gender-based violence, particularly image-based sexual abuse, quantitative surveys—which to the best of our knowledge have not yet been undertaken in Fiji—could help to better establish prevalence and patterns of victimization and perpetration, while interviews and participatory research with young people could provide a rich exploration of how to build prevention programs and initiatives that challenge the cultural norms and gendered expectations we identified that hinder responses to image-based sexual abuse, and other forms of technology-facilitated gender-based violence. Victim-survivors’ experiences of shame and silencing in relation to technology-facilitated gender-based violence, including image-based sexual abuse have been documented in western contexts, but remain underexplored in Pacific settings.

Building on our study findings relating to faith and ethnicity, and given the limited number of gender diverse participants recruited, future research should seek to interrogate how intersecting identities, such as gender, sexuality, ethnicity, and faith compound vulnerability and influence perceptions of responsibility in image-based sexual abuse cases. Culturally led approaches are essential, involving youth voices and indigenous knowledge systems to ensure that research design and interventions are contextually relevant and socially acceptable. Our findings show common harms and gendered forms of victim-blaming with those identified in Western research (Flynn et al., 2023a; Henry et al., 2020), but also highlight Fiji’s distinct patriarchal dynamics, where women targeted by image-based sexual abuse face compounded stigma, as their bodies and reputations are treated as family capital (George, 2025; OHCHR, 2013). Integrating these perspectives positions the Fijian experience within global conversations on technology-facilitated gender-based violence, while foregrounding the cultural and structural factors that shape its local realities. By further embedding these dimensions which emerged from our study in mixed-method approaches, future research can seek to produce evidence that informs policy and practice and provides strategies that empower young people to challenge entrenched norms.

While our focus groups provided rich qualitative insights into young people’s experiences and perceptions of image-based sexual abuse and victim-blaming in Fiji, several limitations should be acknowledged. First, the sample size does not fully represent the broader youth population across Fiji’s geographic, ethnic, and socioeconomic contexts. Second, the group setting may have influenced participants’ willingness to disclose sensitive or personal experiences due to social desirability bias or fear of judgment, particularly in a cultural context where discussions of sexuality and abuse are often stigmatized (Naz, 2014). Third, while efforts were made to ensure trauma-informed training and diversity of ethnicity, faith and gender among the facilitators, the findings were likely shaped by the dynamics of the specific focus groups and facilitators, which may have affected the depth and direction of conversations. Finally, while the study aimed to capture a range of perspectives and sought three diverse locations across Fiji (Labasa, Lautoka, and Suva), it may have missed voices from more marginalized or rural communities. Notably, we had no participants identify as gender diverse, and only a minority identified as same sex attracted, bisexual or preferred not to disclose their sexuality. As such, future research should also seek to broaden representation and deepen understanding of these issues among marginalized communities, such as targeting gender and sexuality diverse participants, to ensure their voices are included.

### Conclusion

Overall, the focus group data indicate a systemic pattern of gendered double standards faced by women victim-survivors of image-based sexual abuse in Fiji, with victim-blaming and moralistic narratives contributing to their silencing, the obstruction of justice, and diminishing perpetrator accountability. As the first empirical study in Fiji to examine young people’s attitudes toward image-based sexual abuse, this research demonstrates how patriarchal norms, communal values, and sexual taboos intersect to shape responses to victim-survivors, highlighting both shared and important distinctions from Western contexts, underscoring the need for local, culturally grounded interventions. The findings point to the importance of context-specific education and awareness initiatives that challenge harmful gender norms and engage community structures, including partnerships with religious and traditional leaders and the development of confidential, empowering support networks. A holistic approach that combines community engagement, sustained public education, and culturally sensitive institutional responses is essential for reducing victim-blaming and improving pathways to justice for victim-survivors.

## Data Availability

Our ethical approval does not permit the qualitative data to be shared beyond the research team.
